# Comparative-high resolution melting: a novel method of simultaneous screening for small mutations and copy number variations

**DOI:** 10.1007/s00439-013-1393-1

**Published:** 2013-11-15

**Authors:** Pawel Borun, Lukasz Kubaszewski, Tomasz Banasiewicz, Jaroslaw Walkowiak, Marzena Skrzypczak-Zielinska, Marta Kaczmarek-Rys, Andrzej Plawski

**Affiliations:** 1 Institute of Human Genetics, Polish Academy of Sciences, Strzeszynska 32, 60-479 Poznan, Poland; 2 Fourth Clinical Hospital, University of Medical Sciences, Poznan, Poland; 3 Department of General, Gastroenterological and Endocrinological Surgery, University of Medical Sciences, Poznan, Poland; 4 Department of Pediatric Gastroenterology and Metabolic Diseases, University of Medical Sciences, Poznan, Poland

## Abstract

Efficient and cost-effective screening for DNA sequence changes, both small mutations and copy number variations (CNVs), is a crucial aspect for routine genetic diagnostics as well as for basic research. In this study we present a development and evaluation of comparative-high resolution melting (C-HRM), a new approach for the simultaneous screening of small DNA changes and gene CNVs. In contrast to other methods, relative quantification in C-HRM is based on the results obtained during the melting process and calculations of the melting peak height ratio in the multiplex reaction. Validation of the method was conducted on DNA samples from 50 individuals from Duchenne muscular dystrophy (DMD) families, 50 probands diagnosed with familial adenomatous polyposis and a control group of 36 women and 36 men. The results of analyses conducted on fragments of the DMD and APC genes correspond completely (100 %) with the results of previous studies. C-HRM sensitivity in CNV detection was assessed through the analysis of mixed DNA samples with different proportions of a deletion carrier and wild type control. The results are presented as a linear regression with *R* ^2^ of 0.9974 and imply the capability of the method to detect mosaics. C-HRM is an attractive and powerful alternative to other methods of point mutations and CNV detection with 100 % accuracy in our studied group.

**Electronic supplementary material:**

The online version of this article (doi:10.1007/s00439-013-1393-1) contains supplementary material, which is available to authorized users.

## Introduction

Hereditary diseases are conditioned by small scale mutations (including point mutations and sequence changes concerning only few nucleotides such as small insertions, deletions, inversions) and the rearrangements of larger genome fragments known as copy number variations (CNVs) (Arlt et al. 2012; Beckmann et al. 2008; Hastings et al. 2009; Henrichsen et al. 2009). One of the challenges of contemporary molecular studies is the efficient and cost-effective detection of both types of changes in the genetic material (Ladabaum et al. 2011). The methods that are currently, widely used require separate analyses to detect small mutations and large rearrangements. A method enabling a simultaneous analysis of both of these types of mutations would provide new possibilities for studies of molecular predisposition to hereditary diseases. Here we present comparative-high resolution melting (C-HRM), a new approach to simultaneous screening for small mutations and CNVs.

To establish and evaluate the C-HRM method we have used DNA from two groups: familial adenomatous polyposis (FAP, MIM 175100) patients and families with diagnosed cases of Duchenne muscular dystrophy (DMD, MIM 310200). Both these disorders are characterized by a significant percentage of CNV mutations in the disease-causing gene. DMD is caused by mutation in the dystrophin gene (*DMD*, MIM 300377) and belongs to X-linked disorders (Bakker et al. 1987; Oshima et al. 2009; Tuffery-Giraud et al. 2009; White et al. 2006; White and den Dunnen 2006). Changes in the adenomatous polyposis coli gene (*APC*, MIM 61173) are responsible for FAP, an autosomal dominant disease (Aretz et al. 2005; Fang et al. 2011; Groden et al. 1991; Stekrova et al. 2007).

This approach was developed on the basis of the standard high resolution melting (HRM) screening method based on the analysis of amplified DNA fragments during the melting process by the presence of a fluorescent dye, intercalating double stranded DNA, in a reaction mixture (Liew et al. 2004; Wittwer 2009; Wittwer et al. 2003). Our new C-HRM method has the advantage over the standard HRM that besides screening for small mutations it enables, at the same time, the detection of large sequence changes such as amplifications and deletions based on the melting peak height ratio in the multiplex reaction. Measurements of the fluorescence level obtained during the melting of amplified fragments may be expressed as a derivative of the fluorescence change in time, making it possible to present the products in a reaction mixture in the form of peaks. Each peak corresponds to a PCR product (or a product melting domain) present in a reaction mixture, and the peak height represents its relative amount. Our studies showed that it is possible to distinguish samples with large deletion from wild types on the basis of the peak height ratio in a multiplex reaction. We decided to use this observation and employ it in the simultaneous analysis of small mutations and CNVs.

## Materials and methods

### DNA samples

We used DNA samples from 50 individuals from DMD families (where the *DMD* gene mutations were studied) and 50 probands diagnosed with FAP (where the *APC* gene mutations were studied) (Kwiatkowska et al. 1997; Lisiecka et al. 1998; Plawski et al. 2004, 2007; Plawski and Slomski 2008). A control group of 72 randomly selected unaffected individuals (36 women and 36 men) from the population was also examined. The studies were approved by the local Ethics Committee of the Poznan University of Medical Science and performed after obtaining written informed consent from all patients and control individuals.

DNA samples of all the patients involved in the verification of the method had been previously tested for the presence of small mutations using screening methods such as single strand conformation polymorphism (SSCP), heteroduplex analysis (HA), high resolution melting (HRM), direct sequencing, and also multiplex ligation probe-dependent amplification (MLPA) to detect CNVs (Schouten et al. 2002; Zielenski et al. 2002). Our study included patients whose gene fragments (or entire genes) had undergone large rearrangements as well as those in which small mutations had been detected.

All patients’ samples were blinded for the purpose of method evaluation. Analyses were performed on groups of 16 samples, out of whom 12 were randomly selected patients and the remaining 4 were control wild type samples. Each sample was analysed in this manner in three separate analyses.

The validation of the C-HRM method for the *DMD* gene involved the analysis of exons 9, 45 and 49. The validation for the *APC* gene included an amplicon for the fragment of exon 15 including nucleotides 2802–2805 (which is one of the hot spots for *APC* gene mutations) and the amplicons covering parts of exons 9 and 14.

### C-HRM primers

We designed sets of primers for a simultaneous amplification of a reference fragment (with an unchanged number of copies) and a target fragment (with the *DMD* or *APC* gene fragments as its template). Designed primers for the *DMD* gene (exons 9, 45 and 49) and the *APC* gene (fragments of exons 9, 14 and 15) include large rearrangements and also small sequence changes detected in our group of patients. The conserved noncoding sequences of the albumin gene (*ALB* MIM 103600) localized at 4q13.3 and the lactate dehydrogenase B gene (*LDHB* MIM 150100) localized at 12p12.1 were used as templates for reference fragments. Primers were designed using the Primer3plus (www.bioinformatics.nl/primer3plus/) software. The melting temperature of all primers was in the range of 58.0–62.3 °C (Table 3).

Subsequently, the primer pairs were selected for a multiplex reaction, with one of the products including the target fragment of the studied gene and the second one as a reference. Amplicons were paired in respect of their melting temperature ranges (no overlaps between the amplicons) and lack of non-specific interactions between primers that could impair the amplification efficiency. Attention was also paid to the size of each product (length of both amplicons was similar), since smaller amplicons tend to amplify with higher efficiency. Sequences of primers and matched pairs are collated in Table 3.

### Assay design

The products were amplified using the type-it HRM kit (Qiagen) on the DNA templates at a concentration of 50 ng/μl diluted in AE buffer (Qiagen). The analysis was performed on a Rotor-Gene^®^ Q equipment (Qiagen). PCR reactions were carried out for the 30 cycles (with a 5 min preincubation at 95 °C) of 95 °C for 10 s, 55 °C for 30 s and 72 °C for 10 s, the products were then melted and PCR was continued to the 40th cycle in the same conditions followed by another melting process. The first melting analysis was performed from 70 °C to 90 °C by raising the temperature by 0.3° at each step after which the second one, designed to detect small changes in the sequence, was carried out with higher resolution raising the temperature by 0.1° at each step.

### Data interpretation and presentation of the results

**Fig. 1:**
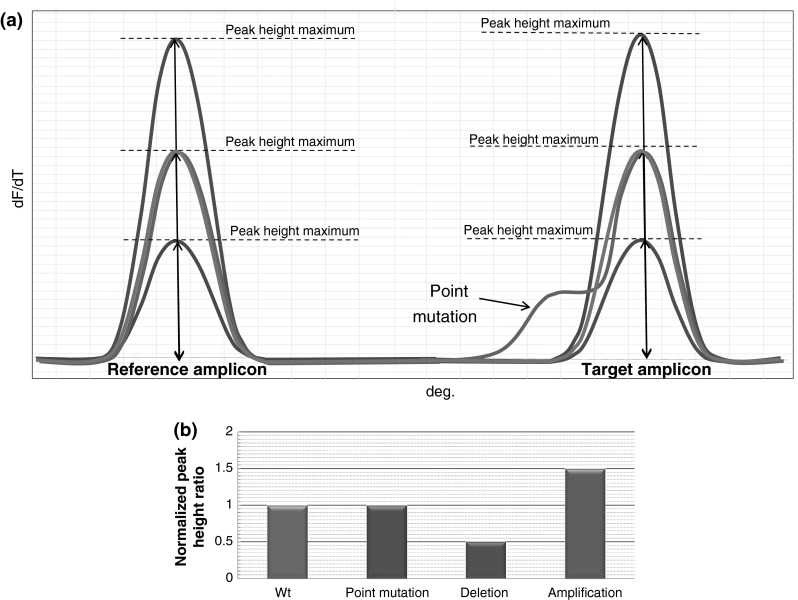
Conceptual graph presenting an outline of the method. **a** Melting profile containing examples of possible genotypes. *Green curve* represents wild type sample, *blue curve* represents sample with amplification of the target amplicon, *red curve* represents a sample with deletion of the target amplicon, *purple curve* represents a sample with point mutation in the target amplicon. **b** Graph results. *Columns* represent normalised peak height ratio of the target amplicon to the reference amplicon calculated for each sample (colour figure online)

The outline of the method is presented in Fig. 1. The result of the first DNA melting process (after the 30th cycle) of wild type samples is a characteristic pattern of a peak height corresponding to both the reference amplicon and the analysed amplicon (in Fig. 1a, green curve). In the case of samples with rearrangements the peak pattern is distorted. When one of the alleles of the analysed fragment undergoes deletion, its peak decreases and, at the same time, the peak of the reference amplicon increases (Fig. 1a, red curve). The situation is reversed in the case of amplification, with the peak of the reference amplicon decreasing and the peak of the analysed amplicon increasing (Fig. 1a, blue curve). In order to observe these differences, the results are compared with DNA samples with an unchanged copy number of templates for each of the amplified fragments.

To present the results in figures and to mitigate the influence of differences in the amplification efficacy of particular samples, the highest values of peaks are determined, followed by calculating the ratio of the analysed peak to the reference peak in each reaction. Subsequently, the ratios obtained are divided by the average value of the peak ratio of all 4 control samples with an unchanged copy number which allows the results to be normalized. The assumption is made that the peak height ratio should be 0.5× for samples with deletion (*n* copies) and 1.5× for samples with duplication (3n copies) compared to the peak height ratio for wild type samples (2*n* copies). The comparison of the ratio obtained for the unknown samples with the ratio of samples with an unchanged number of copies allows samples with possible rearrangements of the analysed gene to be determined (Fig. 1b).

For DNA samples with large rearrangements (both large deletions and large amplifications), the standard peak height ratio observed in the control sample (with a normal number of copies for both amplicons) is distorted. A clear difference can be observed in the peak ratio for samples in which the analysed fragment undergoes deletion or amplification. The difference stems from the change in the initial amount of the template of the analysed gene compared with the amount of the reference amplicon template. The analysis should preferably be performed during the exponential phase of PCR when the efficiency depends mostly on the initial amount of template and when no reagent in the reaction mixture was depleted. Therefore, data for the melting peak height ratio calculations are obtained after the 30th cycle.

Detection of small sequence changes was performed as in the standard HRM. The area of analysis was narrowed only to the target amplicons. Performing a full PCR reaction (up to the 40th cycle) enables a greater amount of PCR products to be achieved, which is beneficial for the detection of small mutations.

### C-HRM sensitivity assessment

The sensitivity of the method was assessed through the analysis of mixed DNA samples with different proportions of a deletion carrier (C) and wild type control (W). The proportions in each reaction were as follows: C 1:W 0; C 0.75:W 0.25; C 0.5:W 0.5; C 0.25:W 0.75; C 0:W 1. The assessment was based on the assay for the *DMD* exon 45. All samples were analysed in triplicates in the presence of four wild type controls for normalization.

## Results

The study enabled us to demonstrate the usefulness of the new C-HRM method in the detection of previously identified small mutations in corresponding amplified fragments with a simultaneous detection of their CNVs (Tables 1, 2). The mean of all normalized peak height ratios for 72 wild type control samples was 0.9944 with standard error of the mean of 0.090033. Assuming that ratios for samples with deletion (*n* copies) should be 0.5× and for samples with duplication (3*n* copies) should be 1.5×, we adopted the standard deviation from the control samples and created an approximate scale to assign the examined sample to one of the groups with: ratio below 0.60 indicating a deletion, ratio between 0.9 and 1.1 indicating the unchanged copy number in relation to the control sample, and ratio above 1.4 indicating an amplification. Obtaining equivocal values (outside the adopted ranges) for any of the samples may be caused by an inappropriate reaction design (unspecific primer binding etc.), the poor quality of a sample (degraded DNA, presence of inhibitors etc.) or pipetting errors. However, it may also indicate possible mosaics. If subsequent analyses give similar results, further investigation using other quantitative methods should be applied.

**Table 1:** Results for the DMD group

DMD patient no.	Gender	Previous results for the *DMD* gene	C-HRM results for exon 9 of the *DMD* gene	C-HRM results for exon 45 of the *DMD* gene	C-HRM results for exon 49 of the *DMD* gene	Correspondence of the results
D1	Male	Deletion of exons 45–48	WT	Deletion of exon 45/no PCR product	WT	Full
D2	Male	Deletion of exons 45–48	WT	Deletion of exon 45/no PCR product	WT	Full
D3	Male	Deletion of exons 45–52	WT	Deletion of exon 45/no PCR product	Deletion of exon 49/no PCR product	Full
D4	Male	Deletion of exons 45–50	WT	Deletion of exon 45/no PCR product	Deletion of exon 49/no PCR product	Full
D5	Female	Deletion of exons 45–48	WT	Deletion of exon 45	WT	Full
D6	Female	Deletion of exons 45–48	WT	Deletion of exon 45	WT	Full
D7	Female	Deletion of exons 45–52	WT	Deletion of exon 45	Deletion of exon 49	Full
D8	Female	Deletion of exons 45–52	WT	Deletion of exon 45	Deletion of exon 49	Full
D9	Female	Deletion of exons 45-52	WT	Deletion of exon 45	Deletion of exon 49	Full
D10	Female	Deletion of exon 45	WT	Deletion of exon 45	WT	Full
D11	Male	Substitution c.6671C>T	WT	Hemizygous Substitution c.6671C>T	WT	Full
D12	Female	Substitution c.6671C>T	WT	Heterozygous Substitution c.6671C>T	WT	Full
D13	Female	Small deletion c.6574_6578delTGGCA	WT	Heterozygous c.6574_6578delTGGCA	WT	Full
D14	Male	Small deletion c.6574_6578delTGGCA	WT	Hemizygous c.6574_6578delTGGCA	WT	Full
D15	Male	Deletion of exons 49–52	WT	WT	Deletion of exon 49/no PCR product	Full
D16	Male	Deletion of exons 48–50	WT	WT	Deletion of exon 49/no PCR product	Full
D17	Female	Amplification of exons 8–10	Amplification of exon 9	WT	WT	Full
D18	Female	Amplification of exons 8–10	Amplification of exon 9	WT	WT	Full
D19	Female	Amplification of exons 5–10	Amplification of exon 9	WT	WT	Full
D20	Male	Amplification of exons 5–10	Amplification of exon 9	WT	WT	Full
D21–50	Female/male	WT	WT	WT	WT	Full

*WT* wild-type

**Table 2:** Results for the APC group

APC patient no.	Previous results for the *APC* gene	C-HRM results for exon 9 of the *APC* gene	C-HRM results for exon 14 of the *APC* gene	C-HRM results for exon 15 of the *APC* gene	Correspondence of the results
A1	Deletion of exons 11-14	WT	Deletion of exon 14	WT	Full
A2	Deletion of whole APC gene	Deletion of exon 9	Deletion of exon 14	Deletion of exon 15	Full
A3	Deletion of whole APC gene	Deletion of exon 9	Deletion of exon 14	Deletion of exon 15	Full
A4	c.1100_1101delCT	c.1100_1101delCT	WT	WT	Full
A5	c.1100_1101delCT	c.1100_1101delCT	WT	WT	Full
A6	c.1879_1882delAACA	WT	c.1879_1882delAACA	WT	Full
A7	c.1744G>T	WT	c.1744G>T	WT	Full
A8	c.1779G>A	WT	c.1779G>A	WT	Full
A9	c.1829delA	WT	c.1829delA	WT	Full
A10	c.2932C>T	WT	WT	c.2932C>T	Full
A11	c.2805C>G	WT	WT	c.2805C>G	Full
A12	c.2802–2805delTTAC	WT	WT	c.2802–2805delTTAC	Full
A13	c.2805C>A	WT	WT	c.2805C>A	Full
A14	c.2802–2805delTTAC	WT	WT	c.2802–2805delTTAC	Full
A15	c.2802–2805delTTAC	WT	WT	c.2802–2805delTTAC	Full
A16	c.2805C>G	WT	WT	c.2805C>G	Full
A17	c.2805C>G	WT	WT	c.2805C>G	Full
A18–50	WT	WT	WT	WT	Full

*WT* wild-type

**Fig. 2:**
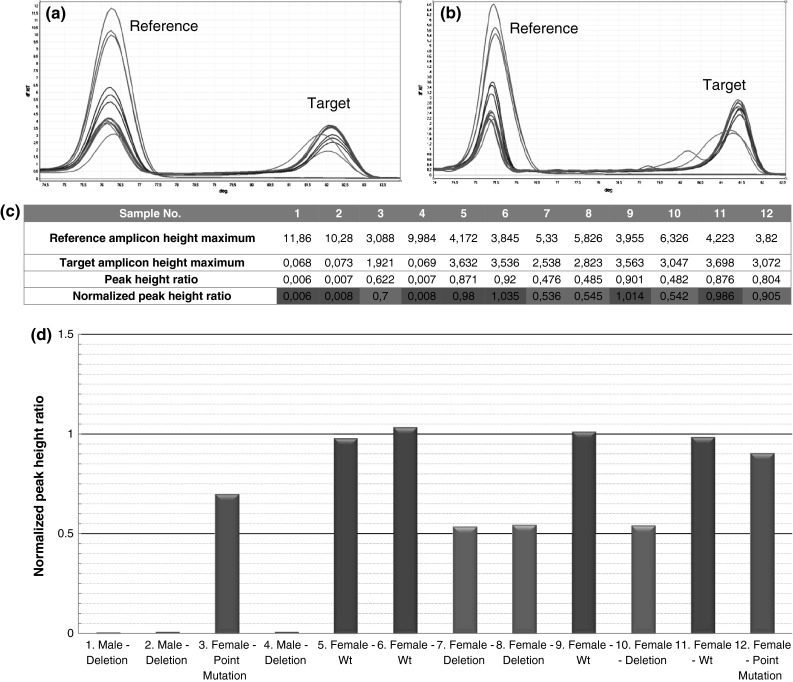
The examination of the *DMD* gene exon 45. An example of detection of small mutations and CNVs present in exon 45. **a** Melting profile of examined samples after the 30th cycle. **b** Melting profile of examined samples after the 40th cycle. **c** Peak height ratio calculations based on the results from melting after the 30th cycle. **d** Graph results. *Blue columns* represent samples with deletion of 1 allele, *red columns* represent hemizygotes with deletion (resulting in a lack of amplification of the target amplicon), *green columns* represent wild types, *pink columns* represent samples with small mutations (colour figure online)

**Fig. 3:**
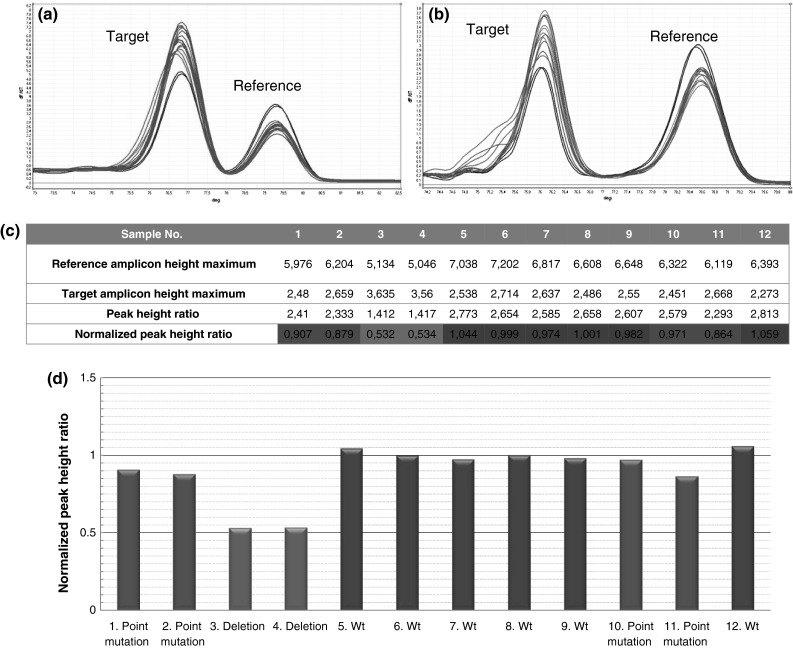
The examination of the *APC* gene exon 15. An example of detection of small mutations and CNVs present in a fragment of exon 15. **a** Melting profile of examined samples after the 30th cycle. **b** Melting profile of examined samples after the 40th cycle. **c** Peak height ratio calculations based on the results from melting after the 30th cycle. **d** Graph results. *Blue columns* represent samples with deletion of 1 allele, *green columns* represent wild types, *pink columns* represent samples with small mutations (colour figure online)

The interpretation of the results of the analyses for genes located on the sex chromosomes (including the *DMD* gene) should be carried out with respect to an individual’s gender. This is caused by the difference in the base copy number of the gene for men and women. In the case of the *DMD* gene studies an unaffected male has one copy of the *DMD* gene, a male duplication carrier has two copies of the amplified fragment (the result of analyses is the same as in the case of healthy women), while a male with a deletion of the studied fragment is devoid of any allele (hence the lack of amplification). Figures 2 and 3 present examples of the results of individual analyses for exon 45 of the *DMD* gene and exon 15 of the *APC* gene. Example results for other exons are included in the Supplementary Material.

### The *DMD* gene studies

Among the 50 examined patients the results were entirely consistent (100 %) with those that had been obtained previously using other methods. All the mutations detected by previous methods were observed and there were no false positive results. In addition, all genotypes of the common intronic polymorphism rs1379871 in the amplicon for exon 49 were also distinguished. Detailed information on the types of the detected mutations is given in Table 1. An example result for exon 45 is presented in Fig. 2. Analysis of 72 unaffected individuals from the control group did not give any false positive results. In addition, the presence of the *DMD* gene on the X chromosome allowed the gender of each of the controls to be determined on the basis of the relative copy number (on the same principle as for a CNV mutation carrier).

### The *APC* gene studies

The results of analyses conducted on fragments of exons 9, 14, 15 of the *APC* gene correspond completely (100 %) with the results of previous studies. All the previously identified mutations in these fragments, both small changes and CNV, were observed during C-HRM. Analysis of the 72 unaffected individuals from the control group did not result in obtaining any false positive results. Detailed information on the types of detected mutations is given in Table 2. An example result for a fragment of exon 15 is presented in Fig. 3.

### C-HRM sensitivity

**Fig. 4:**
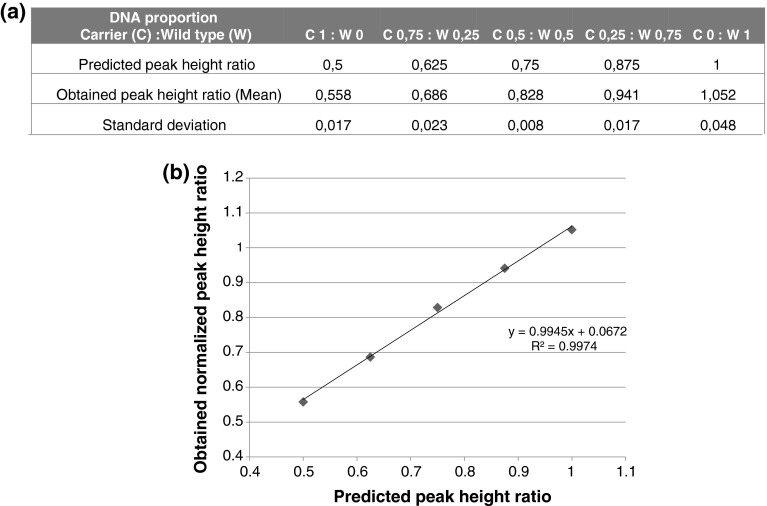
Sensitivity of C-HRM in CNV detection. The sensitivity of the C-HRM method was assessed by mixing different proportions of the DNA from female deletion carrier of the *DMD* exon 45 and wild type control (**a**). The results are presented as a linear regression with *R* ^2^ of 0.9974 (**b**). Taking into consideration the scale we adopted, theoretically it could be possible to distinguish a sample with only 17.3 % of DNA with the deletion

The C-HRM analysis of different proportions of the mutation carrier and wild type control demonstrated the level of sensitivity of the method. The results are presented as a linear regression with *R* ^2^ of 0.9974 (Fig. 4). Taking into consideration the scale we adopted, theoretically it could be possible to distinguish a sample containing only 17.3 % of DNA with the deleted allele which implies that C-HRM has the capability to detect mosaics.

## Discussion

The detection of mutations in genetic material is a major challenge for both genetic diagnostics and molecular genetic studies. Screening methods allow the costs of analysis to be decreased by reducing the amount of sequencing. Commonly used methods of screening for mutations enable the detection of only one type of mutation in a single analysis. The aim of our study was to validate the invented method for the simultaneous detection of CNVs and small mutations.

The comparative-HRM method we developed allows a simultaneous detection of small mutations and CNVs in the analysed fragments. This new approach has the advantage over previously employed methods for mutation screening in that it makes it possible to detect both types of mutation during a single analysis. Screening methods such as HRM, HA, SSCP and direct PCR product sequencing fail in the case of CNVs. Our C-HRM method may turn out to be an attractive alternative to the MLPA and quantitative-PCR (qPCR) that are currently, widely used in diagnostics for detecting CNVs, especially taking into consideration its simplicity and easy application. In contrast to qPCR methods in which quantification is based on amplification curve data, our method uses peak height ratio calculations obtained during melting analysisto determine the relative copy number. This opens up the possibility of its application in cases where the qPCR fails due to e.g. low amplification efficiency.

The C-HRM method does not require any other reagents apart from those used in the standard HRM and the analysis takes only slightly more time, being extended by the time required for the additional melting process, however, it does not need any further processing and separation of the PCR products as in MLPA. Moreover, there is no need for any special reagents (unlike the case of MLPA) or for a significant number of samples with known parameters to create a standard curve (unlike in qPCR), and the method itself may be employed in every laboratory equipped with an HRM device. The costs of the analysis are almost equal to those for the standard HRM since they are increased only by the cost of additional primers. C-HRM allows the study of larger amplicons (in our assay up to 249 bp) than those recommended for qPCR (80–150 bp) which also reduces expenses and number of analyses (D’Haene et al. 2010). In addition, the method seems to be unaffected by the fluctuations of the template concentration (the amount of template has no influence on the peak ratio as both fragments are amplified in one reaction tube).

A clear advantage of the MLPA method over C-HRM is that MLPA can detect copy number alteration of multiple (>20) loci in a single assay (Schouten et al. 2002). However, it does not allow the screening of these loci for small mutations and their separate screening must be applied. Furthermore, MLPA requires additional processing and the use of capillary electrophoresis equipment which is unnecessary for C-HRM analysis. We are confident that in the future it will be possible to design specific assays for whole coding sequences of most disease causing genes enabling C-HRM performance.

C-HRM is not only useful in the detection of CNVs but is also equally effective as a standard HRM in the screening for small mutations. Our observations indicate that an additional reference product does not decrease the effectiveness of the HRM method in detecting small mutations. In the analysed fragments of genes, in multiplex reactions using the reference fragments, we observed all the previously detected small mutations (Tables 1, 2). Although we observed them during the melting process after 30 and after 40 cycles, a larger amount of the product is more beneficial for small mutations that are more difficult to distinguish after the 30th cycle. Therefore, we prefer performing additional amplification cycles followed by an analysis of samples with respect to the presence of small sequence changes.

During the validation of the method for each of the tested DNA sample a series of three separate analyses were performed. Fluctuations in the peak height ratios in subsequent analyses of each sample ranged between 5 and 10 %. However, they did not affect the consistency of the results obtained. All three repeated analyses for each sample indicated consistent results and each time it was possible to easily distinguish a sample with deletion/duplication from a sample with an unchanged number of copies. Most probably, these fluctuations are not due to the imperfection of the method but are rather associated with minimal differences in the composition of each of the reaction mixtures (including the concentration of each primer) and the amount of the initial template in each repetition, both resulting from the inaccuracy of manual pipetting techniques. These results may suggest 100 % specificity of the method. Nevertheless, it must be taken into account that the development of the methodology was conducted on high quality DNA samples specially prepared for the requirements of the HRM analysis. C-HRM (as other quantification analyses) is sensitive to the degree of degradation and the quality of the genetic material used for the research. Despite the fact that in our group of patients we did not obtain any false positive results, their occurrence in other groups cannot be ruled out. In the case of an analysis of a larger cohort of samples with various degrees of quality we do not exclude the possibility of obtaining false results.

The key factor for successful C-HRM analysis, as for qPCR, is the specificity of the designed primers (D’Haene et al. 2010). Apart from high specificity, primers must be able to amplify in a multiplex reaction, therefore a uniform annealing temperature is required. In addition, there has to be lack of non-specific interactions between all 4 primers, since non-specifically binding primers may lower the PCR efficiency. Amplification efficiency is higher for shorter products, thus both amplicons should be of a similar size. Furthermore, for proper small mutation screening, target and reference melting peaks cannot overlap, thus products must have different melting temperatures. Most likely, it will be difficult to find a universal reference amplicon as a control for all C-HRM analyses. In our study we used reference amplicons based on a two highly conserved gene fragments, and one worked with five of the six studied amplicons (Table 3).

**Table 3:** Sets of primers

Gene	Studied exon			Product length (bp)	Forward primer	Tm (°C)	Final concentration (nM)	Reverse primer	Tm (°C)	Final concentration (nM)
*APC*	9	Target amplicon	APC	249	GCCCACAGGTGGAAATGG	62.3	172	GAATGATGTTGTGGAGTGCTG	59.2	172
		Reference amplicon	LDHB	252	TGAAAAACTGTTTGGCAGAGTC	59.4	688	GCCTGGTACAGAGCATTGTG	59.3	688
	14	Target amplicon	APC	239	GAAGTTAATGAGAGACAAATTCCAA	58.0	172	TCCGTAATATCCCACCTCCA	60.1	172
		Reference amplicon	LDHB	252	TGAAAAACTGTTTGGCAGAGTC	59.4	688	GCCTGGTACAGAGCATTGTG	59.3	688
	15	Target amplicon	APC	180	TCTGCTGCCCATACACATTC	59.7	688	GGATTCAATCGAGGGTTTCA	59.9	688
		Reference amplicon	ALB	196	TCTTGAATGCCTCTTTGCTG	59.1	344	AAACATGCCAGTCCCTGTTC	60.0	344
*DMD*	9	Target amplicon	DMD	228	TATGGTTTTTCCCCCTCCTC	60.1	172	TGGAAGCAGTTCTCTGGTTTG	60.4	172
		Reference amplicon	LDHB	252	TGAAAAACTGTTTGGCAGAGTC	59.4	688	GCCTGGTACAGAGCATTGTG	59.3	688
	45	Target amplicon	DMD	242	CATGGGGCTTCATTTTTGTT	59.8	344	TTCCTATTAGATCTGTCGCCCTA	59.3	344
		Reference amplicon	LDHB	252	TGAAAAACTGTTTGGCAGAGTC	59.4	688	GCCTGGTACAGAGCATTGTG	59.3	688
	49	Target amplicon	DMD	208	TGCACTATATGGGTTCTTTTCC	58.1	172	CCACGTCAATGGCAAATGTA	60.4	172
		Reference amplicon	LDHB	252	TGAAAAACTGTTTGGCAGAGTC	59.4	750	GCCTGGTACAGAGCATTGTG	59.3	750

The results of C-HRM for samples with small mutations may be inaccurate due to the change of shape of the target amplicon peak caused by a different melting behaviour in heterozygous samples (Figs. 2, 3). A similar situation appears in the case of result interpretation for amplicons containing common polymorphisms (as in the amplicon for exon 49 of the *DMD* gene, designed by us, which encompasses rs1379871). However, this does not reduce the detectability of the method. There is no need to calculate ratios for samples with small mutations so we recommend analysing results in terms of small mutations at first, and subsequently calculating peak height ratios for other samples where no small mutations were detected. This approach not only overcomes problems with incorrect results but also reduces the number of samples that require calculations. This also applies to the heterozygous samples, since heterozygosity implies the presence of at least two alleles, thus lack of alteration. Problems with calculations would concern only rare cases of amplifications containing heterozygous polymorphic sites. However, our studies indicate that even such cases can be properly diagnosed due to the smaller impact of the imbalanced alleles on the shape of the peak (66–33 % in the alleles ratio). Nevertheless, to correctly validate the method, for rs1379871 in exon 49 of the *DMD* gene we used two sets of control samples: wild type controls for the normalization of wild type samples and heterozygous controls for the normalization of heterozygous samples. In the future it will also be possible to use the areas of individual peaks instead of their maximum height to calculate ratios of the target amplicon to the reference amplicon. This could increase the accuracy of the method and overcome problems with the calculation for amplicons including polymorphic sites.

Comparative-HRM is a rapid, inexpensive, high throughput method to prescreen for small mutations and large rearrangements in a single run. This novel and reliable approach uses melting peak height ratio calculations instead of an amplification curve for relative quantification. There is no need for electrophoretic separation or additional handling, since the whole analysis is conducted in a close tube system. The method could be a useful tool for simultaneous screening for small DNA mutations and CNVs prior to characterization by sequencing or array-comparative genomic hybridization. It enabled both types of mutations to be revealed rapidly in a large series of samples with 100 % accuracy.

## Electronic supplementary material

Below is the link to the electronic supplementary material.

Supplementary material 1 (PPTX 291 kb)
