# Supplementary material for: Comparative-high resolution melting: a novel method of simultaneous screening for small mutations and copy number variations
Source: Hum Genet. 2013 Nov 15;133(5):535–45. doi: 10.1007/s00439-013-1393-1 (PMC3984413; doi:10.1007/s00439-013-1393-1)
Supplement: Supplementary file 1 — Supplementary material 1 (PPTX 291 kb) [file 439_2013_1393_MOESM1_ESM.pptx]

## Slide 1
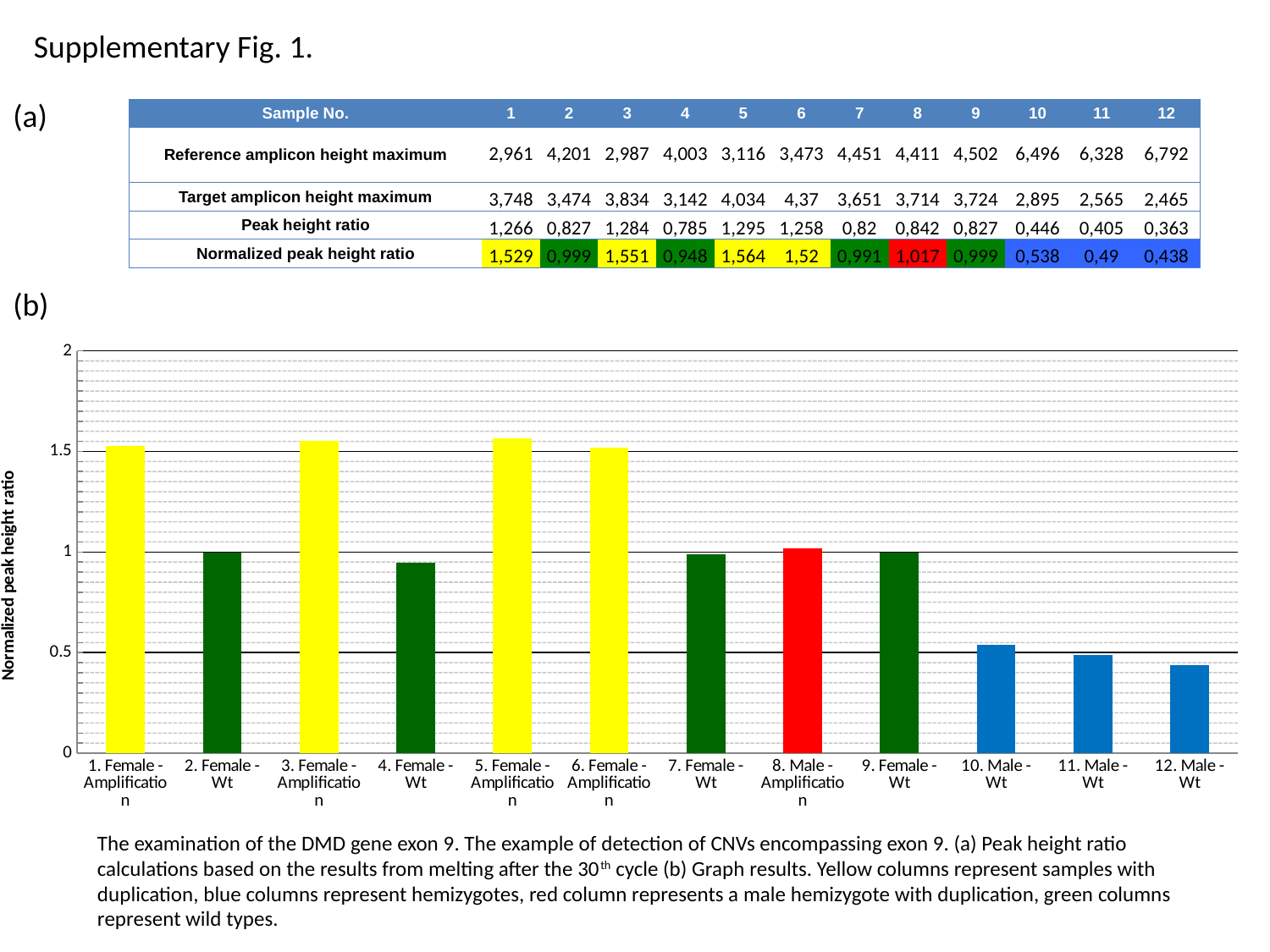

Supplementary Fig. 1.
(a)
| Sample No. | 1 | 2 | 3 | 4 | 5 | 6 | 7 | 8 | 9 | 10 | 11 | 12 |
| --- | --- | --- | --- | --- | --- | --- | --- | --- | --- | --- | --- | --- |
| Reference amplicon height maximum | 2,961 | 4,201 | 2,987 | 4,003 | 3,116 | 3,473 | 4,451 | 4,411 | 4,502 | 6,496 | 6,328 | 6,792 |
| Target amplicon height maximum | 3,748 | 3,474 | 3,834 | 3,142 | 4,034 | 4,37 | 3,651 | 3,714 | 3,724 | 2,895 | 2,565 | 2,465 |
| Peak height ratio | 1,266 | 0,827 | 1,284 | 0,785 | 1,295 | 1,258 | 0,82 | 0,842 | 0,827 | 0,446 | 0,405 | 0,363 |
| Normalized peak height ratio | 1,529 | 0,999 | 1,551 | 0,948 | 1,564 | 1,52 | 0,991 | 1,017 | 0,999 | 0,538 | 0,49 | 0,438 |
(b)
### Chart
| Category | |
|---|---|
| 1. Female - Amplification | 1.5291774617058564 |
| 2. Female - Wt | 0.99901922576254 |
| 3. Female - Amplification | 1.5506493431159474 |
| 4. Female - Wt | 0.9482378881685113 |
| 5. Female - Amplification | 1.5639942730452336 |
| 6. Female - Amplification | 1.52010422897704 |
| 7. Female - Wt | 0.9909481979676608 |
| 8. Male - Amplification | 1.0171887662059322 |
| 9. Female - Wt | 0.9993115228965899 |
| 10. Male - Wt | 0.5383928273076289 |
| 11. Male - Wt | 0.48968591510266996 |
| 12. Male - Wt | 0.4384458461879989 |The examination of the DMD gene exon 9. The example of detection of CNVs encompassing exon 9. (a) Peak height ratio calculations based on the results from melting after the 30th cycle (b) Graph results. Yellow columns represent samples with duplication, blue columns represent hemizygotes, red column represents a male hemizygote with duplication, green columns represent wild types.

## Slide 2
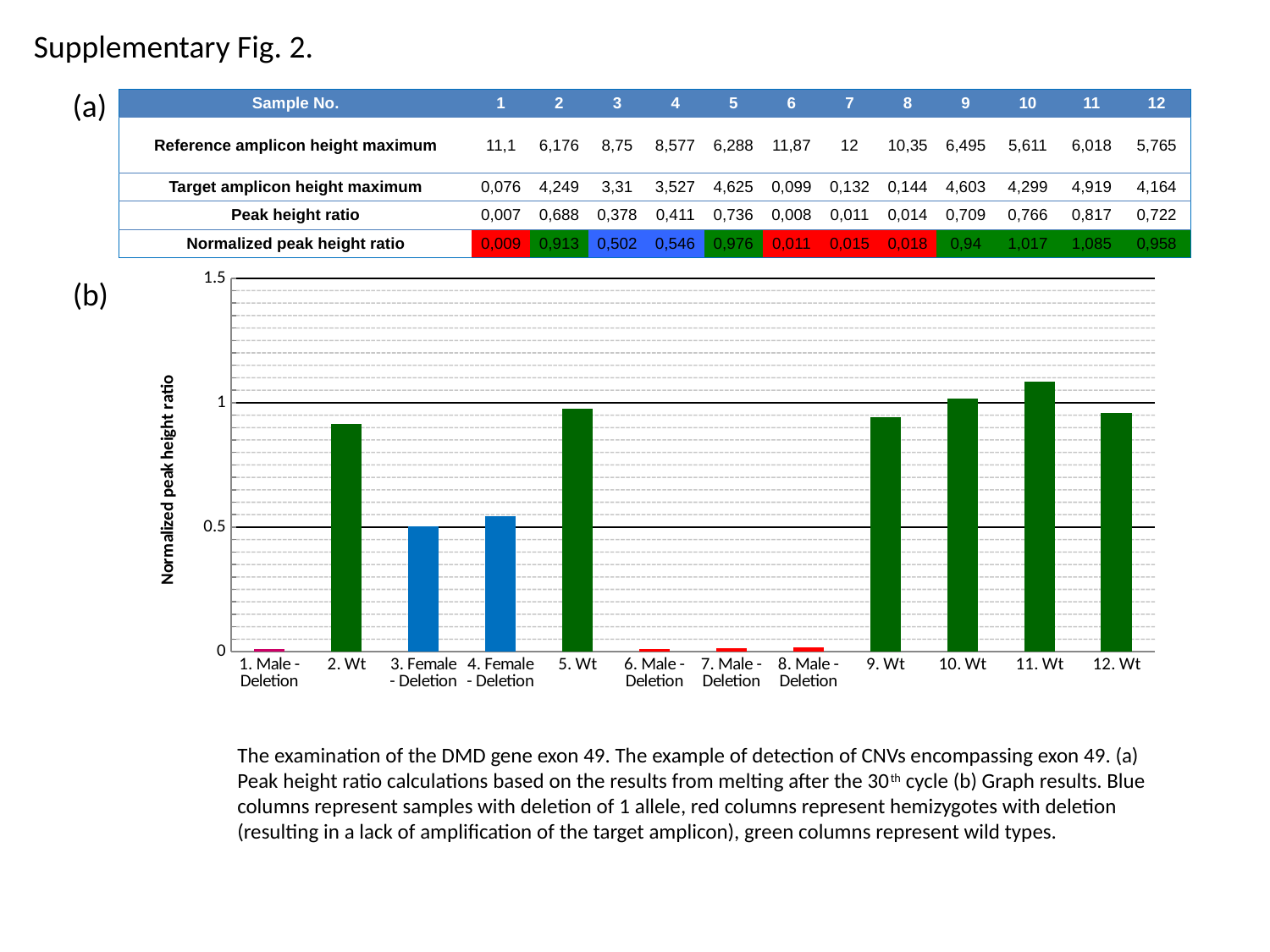

Supplementary Fig. 2.
(a)
| Sample No. | 1 | 2 | 3 | 4 | 5 | 6 | 7 | 8 | 9 | 10 | 11 | 12 |
| --- | --- | --- | --- | --- | --- | --- | --- | --- | --- | --- | --- | --- |
| Reference amplicon height maximum | 11,1 | 6,176 | 8,75 | 8,577 | 6,288 | 11,87 | 12 | 10,35 | 6,495 | 5,611 | 6,018 | 5,765 |
| Target amplicon height maximum | 0,076 | 4,249 | 3,31 | 3,527 | 4,625 | 0,099 | 0,132 | 0,144 | 4,603 | 4,299 | 4,919 | 4,164 |
| Peak height ratio | 0,007 | 0,688 | 0,378 | 0,411 | 0,736 | 0,008 | 0,011 | 0,014 | 0,709 | 0,766 | 0,817 | 0,722 |
| Normalized peak height ratio | 0,009 | 0,913 | 0,502 | 0,546 | 0,976 | 0,011 | 0,015 | 0,018 | 0,94 | 1,017 | 1,085 | 0,958 |
### Chart
| Category | |
|---|---|
| 1. Male - Deletion | 0.009084267876709821 |
| 2. Wt | 0.913 |
| 3. Female - Deletion | 0.5019476014810038 |
| 4. Female - Deletion | 0.5456428795272972 |
| 5. Wt | 0.9759726481090136 |
| 6. Male - Deletion | 0.011069619546726459 |
| 7. Male - Deletion | 0.014593476105205009 |
| 8. Male - Deletion | 0.018454095936362283 |
| 9. Wt | 0.9403732401242544 |
| 10. Wt | 1.016636315061417 |
| 11. Wt | 1.0845837212553806 |
| 12. Wt | 0.9584067235589477 |(b)
The examination of the DMD gene exon 49. The example of detection of CNVs encompassing exon 49. (a) Peak height ratio calculations based on the results from melting after the 30th cycle (b) Graph results. Blue columns represent samples with deletion of 1 allele, red columns represent hemizygotes with deletion (resulting in a lack of amplification of the target amplicon), green columns represent wild types.

## Slide 3
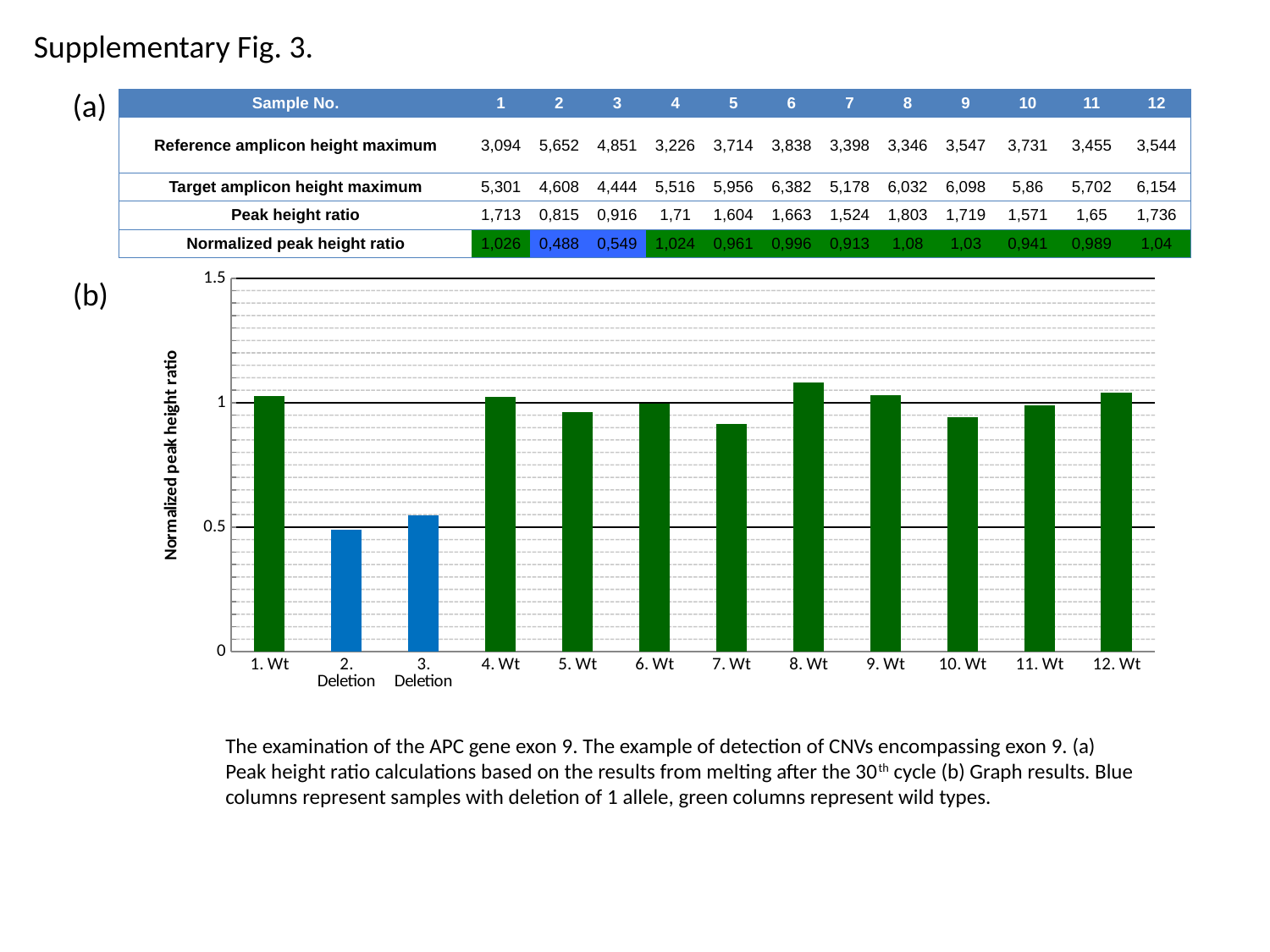

Supplementary Fig. 3.
(a)
| Sample No. | 1 | 2 | 3 | 4 | 5 | 6 | 7 | 8 | 9 | 10 | 11 | 12 |
| --- | --- | --- | --- | --- | --- | --- | --- | --- | --- | --- | --- | --- |
| Reference amplicon height maximum | 3,094 | 5,652 | 4,851 | 3,226 | 3,714 | 3,838 | 3,398 | 3,346 | 3,547 | 3,731 | 3,455 | 3,544 |
| Target amplicon height maximum | 5,301 | 4,608 | 4,444 | 5,516 | 5,956 | 6,382 | 5,178 | 6,032 | 6,098 | 5,86 | 5,702 | 6,154 |
| Peak height ratio | 1,713 | 0,815 | 0,916 | 1,71 | 1,604 | 1,663 | 1,524 | 1,803 | 1,719 | 1,571 | 1,65 | 1,736 |
| Normalized peak height ratio | 1,026 | 0,488 | 0,549 | 1,024 | 0,961 | 0,996 | 0,913 | 1,08 | 1,03 | 0,941 | 0,989 | 1,04 |
### Chart
| Category | |
|---|---|
| 1. Wt | 1.0264538365337406 |
| 2. Deletion | 0.48844114953689777 |
| 3. Deletion | 0.5488386697992196 |
| 4. Wt | 1.0243817246414588 |
| 5. Wt | 0.960759565585363 |
| 6. Wt | 0.996216560476818 |
| 7. Wt | 0.913 |
| 8. Wt | 1.0800337417789698 |
| 9. Wt | 1.0299784994152898 |
| 10. Wt | 0.9409667867129633 |
| 11. Wt | 0.9887376882270359 |
| 12. Wt | 1.0403170256447105 |(b)
The examination of the APC gene exon 9. The example of detection of CNVs encompassing exon 9. (a) Peak height ratio calculations based on the results from melting after the 30th cycle (b) Graph results. Blue columns represent samples with deletion of 1 allele, green columns represent wild types.

## Slide 4
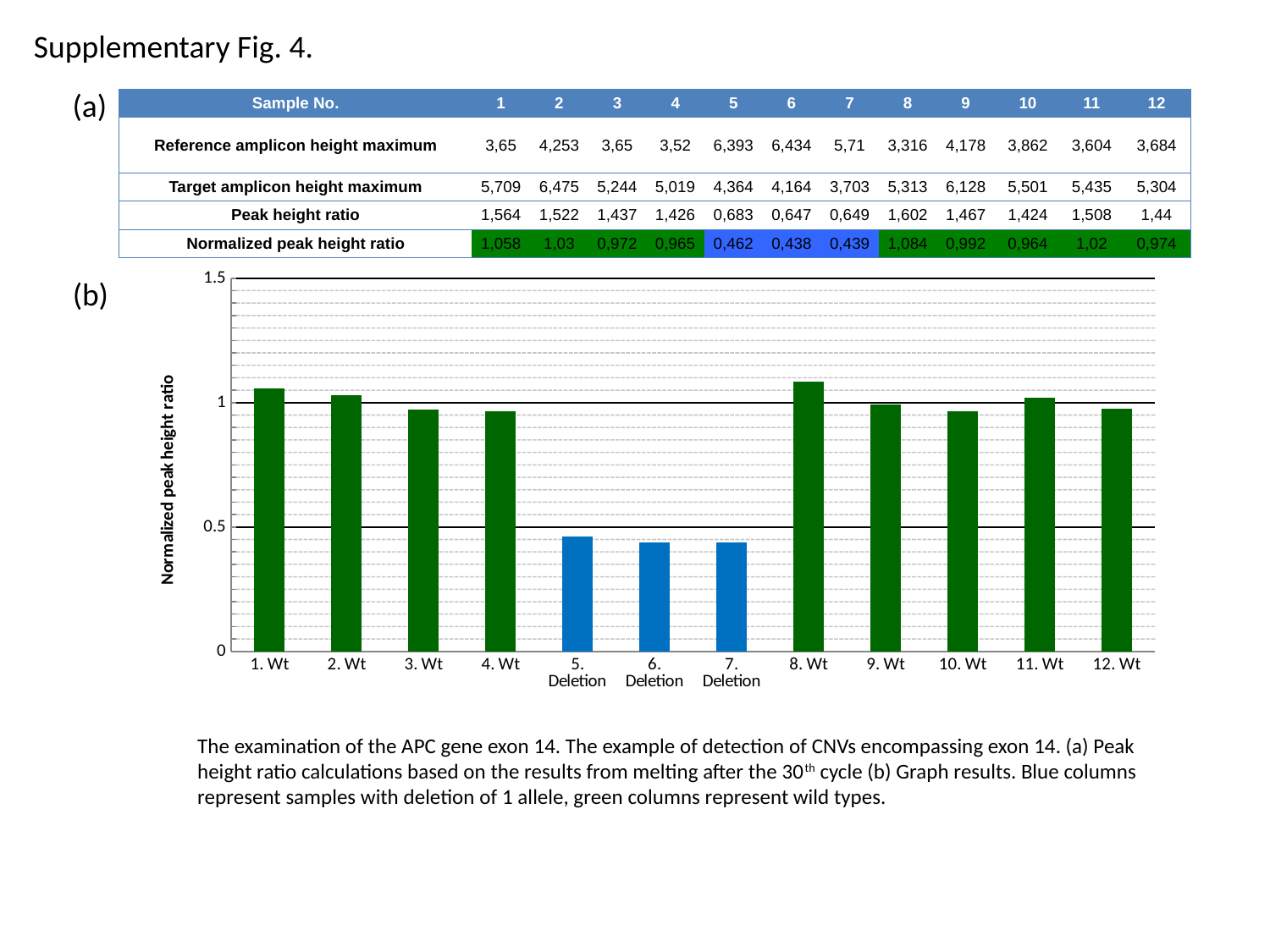

Supplementary Fig. 4.
(a)
| Sample No. | 1 | 2 | 3 | 4 | 5 | 6 | 7 | 8 | 9 | 10 | 11 | 12 |
| --- | --- | --- | --- | --- | --- | --- | --- | --- | --- | --- | --- | --- |
| Reference amplicon height maximum | 3,65 | 4,253 | 3,65 | 3,52 | 6,393 | 6,434 | 5,71 | 3,316 | 4,178 | 3,862 | 3,604 | 3,684 |
| Target amplicon height maximum | 5,709 | 6,475 | 5,244 | 5,019 | 4,364 | 4,164 | 3,703 | 5,313 | 6,128 | 5,501 | 5,435 | 5,304 |
| Peak height ratio | 1,564 | 1,522 | 1,437 | 1,426 | 0,683 | 0,647 | 0,649 | 1,602 | 1,467 | 1,424 | 1,508 | 1,44 |
| Normalized peak height ratio | 1,058 | 1,03 | 0,972 | 0,965 | 0,462 | 0,438 | 0,439 | 1,084 | 0,992 | 0,964 | 1,02 | 0,974 |
### Chart
| Category | Normalized peak height ratio |
|---|---|
| 1. Wt | 1.0581978860321422 |
| 2. Wt | 1.0300163086022982 |
| 3. Wt | 0.9720073067704597 |
| 4. Wt | 0.964659939025847 |
| 5. Deletion | 0.4618274560474034 |
| 6. Deletion | 0.4378540542235668 |
| 7. Deletion | 0.43875018728581816 |
| 8. Wt | 1.083989324359714 |
| 9. Wt | 0.9923160870622391 |
| 10. Wt | 0.9636716583933812 |
| 11. Wt | 1.0202684972184093 |
| 12. Wt | 0.9740552801854407 |(b)
The examination of the APC gene exon 14. The example of detection of CNVs encompassing exon 14. (a) Peak height ratio calculations based on the results from melting after the 30th cycle (b) Graph results. Blue columns represent samples with deletion of 1 allele, green columns represent wild types.
